# Detection of missed fractures of hand and forearm in whole-body CT in a blinded reassessment

**DOI:** 10.1186/s12891-021-04425-z

**Published:** 2021-06-26

**Authors:** S. Kim, L. Goelz, F. Münn, D. Kim, M. Millrose, A. Eisenschenk, S. Thelen, M. Lautenbach

**Affiliations:** 1 Klinik und Poliklinik für Unfall-, Wiederherstellungschirurgie und Rehabilitative Medizin, Universitätsmedizin Greifswald, Greifswald, Germany; 2 Leibniz Institut für Plasmaforschung und Technologie (INP Greifswald), Greifswald, Germany; 3 Institut für Radiologie und Neuroradiologie, Unfallkrankenhaus Berlin, Berlin, Germany; 4 Klinik für Radiologie, Charité - Universitätsmedizin Berlin, Berlin, Germany; 5 Abteilung für Unfallchirurgie und Sporttraumatologie, BG Unfallklinik Murnau, Murnau am Staffelsee, Germany; 6 Abteilung für Hand-, Replantations- und Mikrochirurgie, Unfallkrankenhaus Berlin, Berlin, Germany; 7 Klinik für Orthopädie und Unfallchirurgie, Universitätsklinikum Düsseldorf, Düsseldorf, Germany

**Keywords:** Polytrauma, Fracture, Hand, Missed, Delayed, Late, Diagnosis, Whole-body CT, Fatigue

## Abstract

**Background:**

We examined the visibility of fractures of hand and forearm in whole-body CT and its influence on delayed diagnosis. This study is based on a prior study on delayed diagnosis of fractures of hand and forearm in patients with suspected polytrauma.

**Methods:**

Two blinded radiologists examined CT-scans of patients with fractures of hand or forearm that were diagnosed later than 24 h after admission and control cases with unremarkable imaging of those areas. They were provided with clinical information that was documented in the admission report and were asked to examine forearm and hands. After unblinding, the visibility of fractures was determined. We examined if time of admission or slice thickness was a factor for late or missed diagnoses.

**Results:**

We included 72 known fractures in 36 cases. Of those 65 were visible. Sixteen visible fractures were diagnosed late during hospital stay. Eight more fractures were detected on revision by the radiologists. Both radiologists missed known fractures and found new fractures that were not reported by the other. Missed and late diagnoses of fractures occurred more often around 5 pm and 1 am. Slice thickness was not significantly different between fractures and cases with fractures found within 24 h and those found later.

**Conclusions:**

The number of late diagnosis or completely missed fractures of the hand and forearm may be reduced by a repeated survey of WBCT with focus on the extremities in patients with suspected polytrauma who are not conscious.

**Level of evidence:**

III

**Supplementary Information:**

The online version contains supplementary material available at 10.1186/s12891-021-04425-z.

## Background

Injuries of the hand may be missed in 3.5 to 25% of patients with polytrauma [1, 2]. While there is no definition of ‘missed’, delayed’, or ‘late’, we decided to use the term ‘late diagnosis’ for injuries that were detected 24 h after admission but during hospitalisation as they were eventually found [3–6]. Fractures of the upper extremity may be associated with reduced quality of life [7–9]. A timely treatment has been shown to be beneficial for a return to work [10]. We found that fractures of hand are more often detected in patients in cases with full inclusion of the hand in the whole-body CT (WBCT). This was more often achieved by placing the hands on the abdomen [6]. The ISS did not have an influence on the number of late diagnoses in our sample [6]. The question remained if the fractures are visible for the human eye on retrospection which may also depend on the slice thickness of the WBCT. Other causes for missed or late diagnoses can be fatigue which has been associated with worse diagnostic performance of radiologists [11]. This study is a follow up of a retrospective analysis of patient data on the sensitivity of WBCT for the detection of fractures of hand and/or forearm in intubated patients with suspected polytrauma [6].

### Aim

We wanted to assess how many fractures of hand and forearm, that were diagnosed late, were visible in the WBCT on retrospection. Additionally, the influence of the time of day of the WBCT and slice thickness on the occurrence of late diagnoses was analysed.

## Methods

The study is based on cases that were identified in the previous publication [6]. The sample consisted of patients who were admitted for suspected polytrauma, sedated and ventilated and received a WBCT. Sedated, intubated patients were chosen to avoid the influence of patient related factors like level of consciousness, self-awareness, and pain sensation.

Two radiologists from two different hospitals that did not provide data for the first study were tasked to examine WBCTs for bony injuries of hand or forearm, no further instructions were given. They were presented 44 cases with the clinical data provided on the CT request form. The cases were in random order and consisted of 12 cases with a late (> 24 h after admission) diagnosis of a fracture of the hand and/or forearm, 25 cases with a diagnosis within 24 h, and 7 control cases who had no injury. The control cases were chosen among cases who received additional imaging of the hand and wrist that showed no bony pathology. All fractures that had been diagnosed before discharge and were listed in the discharge papers are named ‘reported fractures’ in our manuscript. Further data after discharge were not available.

Radiologist 1 was a 4th year resident, radiologist 2 had more than 20 years of working experience. They were permitted to use all sequences of the WBCT, including the localiser, and do additional reconstruction if needed. They were asked to list all bony injuries of the upper extremities without further instructions. After blinded reassessment, all reported, and previously unreported fractures were tested for inclusion and visibility in the CT by three of the authors. Discrepancies were solved by majority vote.

Eight fractures were found that have not been reported during hospitalisation. Those missed fractures were added to the number of fractures with diagnosis later than 24 h. As they probably would not have needed a surgical treatment and likely full healing, we decided to not contact the patients. The patient should not be confronted with memories of the traumatic event. This decision was supported by the ethical board of our institution, also considering that there would be no therapeutic consequence by now.

Time of imaging and slice thickness of the axial layers analysed. For the time diagrams, fractures and cases were categorised into diagnosis ‘< 24 h’, ‘> 24 h’, and ‘missed’. Cases with at least one missed fracture were labelled ‘missed’, then cases with at least one late diagnosis were labelled ‘> 24 h’. The other were ‘< 24 h’.

Statistical tests of categorial variables with at least 5 expected cases for each field were performed with Chi-square, and Fisher’s exact for tables that did not meet the requirement. Differences between continuous variables were tested using the unpaired t-test. A *p*-value of ≤0.05 was defined as significant.

The local institutional ethics committee of the University of Greifswald (Ethikkommission an der Universitätsmedizin Greifswald, Greifswald, Germany) approved the study (BB 054/16a) and stated that there are no ethical or legal concerns regarding this study. The decision was based on the Helsinki declaration. The need for consent for the retrospective use of patient data was waived by the institutional ethics committee of the University of Greifswald.

Informed consent was obtained from the radiologists that participated in the study.

## Results

Seventy-two fractures of hand or forearm were reported in the discharge letter of 36 cases. Forty-nine were found within 24 h after admission by WBCT or additional diagnostic imaging. On reassessment of all reported fractures, 65 were visible resulting in 25% (16 of 65) of diagnoses that could have been detected additionally on admission in the initial WBCT (Table 1).

**Table 1:** Reported fractures and their visibility in WBCT

	Patient chart		Study review	
Fracture location	Reported	Reported within 24 h	Area shown	Fracture visible
Ulna	20	16	19	19
Radius	16	14	15	15
Carpus	7	5	7	7
MC	18	15	17	17
Phalanx	11	6	10	7
Total	72	56	68	65

The number of fractures that were known at discharge are shown with the number of fractures that were found within 24 h. On review of all imaging data we determined how many fractures were included in the CT scan area and how many could be recognised

Twenty-four requests forms had clinical data on suspected injuries. A suspected injury of forearm and hands was documented in 13 cases. Eight of those had no injury in the suspected area, of which three were control cases. Five cases had a corresponding injury. Fourteen cases reported the mechanism of injury only, of which four only mentioned ‘traffic injury’. Five request forms gave information that the patient is sedated and intubated only, and one had no entry.

### Blinded reassessment

Both blinded radiologists missed reported fractures and suspected 15 more fractures (Table 2).

**Table 2:** Missed and reported fractures on blinded and unblinded reassessment

	Radiologist 1			Radiologist 2			Unblinded
Fracture location	Missed	New	Confirmed	Missed	New	Confirmed	Total visible
Ulna	7	0	0	3	3	3	22
Radius	4	0	0	3	1	0	15
Carpus	7	1	1	3	4	3	11
MC	2	1	1	3	4	1	18
Phalanx	6	1	0	10	0	0	7
Total	26	3	2	22	12	7	73

The ‘missed’ column shows the number of visible fractures from Table 1 that were not found by each radiologist. ‘New’ are previously not reported fractures of which ‘confirmed’ could be confirmed in the WBCT upon review. The ‘total visible’ column adds the number of visible reported and confirmed new fractures. One metacarpal fracture was described by both radiologists. All other new were only mentioned by one of both

We could confirm eight fractures on unblinded reassessment. One was found by both. Added to the 65 visible fractures that were reported in the discharge documentation, in total 73 fractures were visible in 33 cases and 11% (8/73) were missed. The remaining 11 cases were 7 controls and 4 cases with fractures not visible in WBCT.

**Fig. 1:**
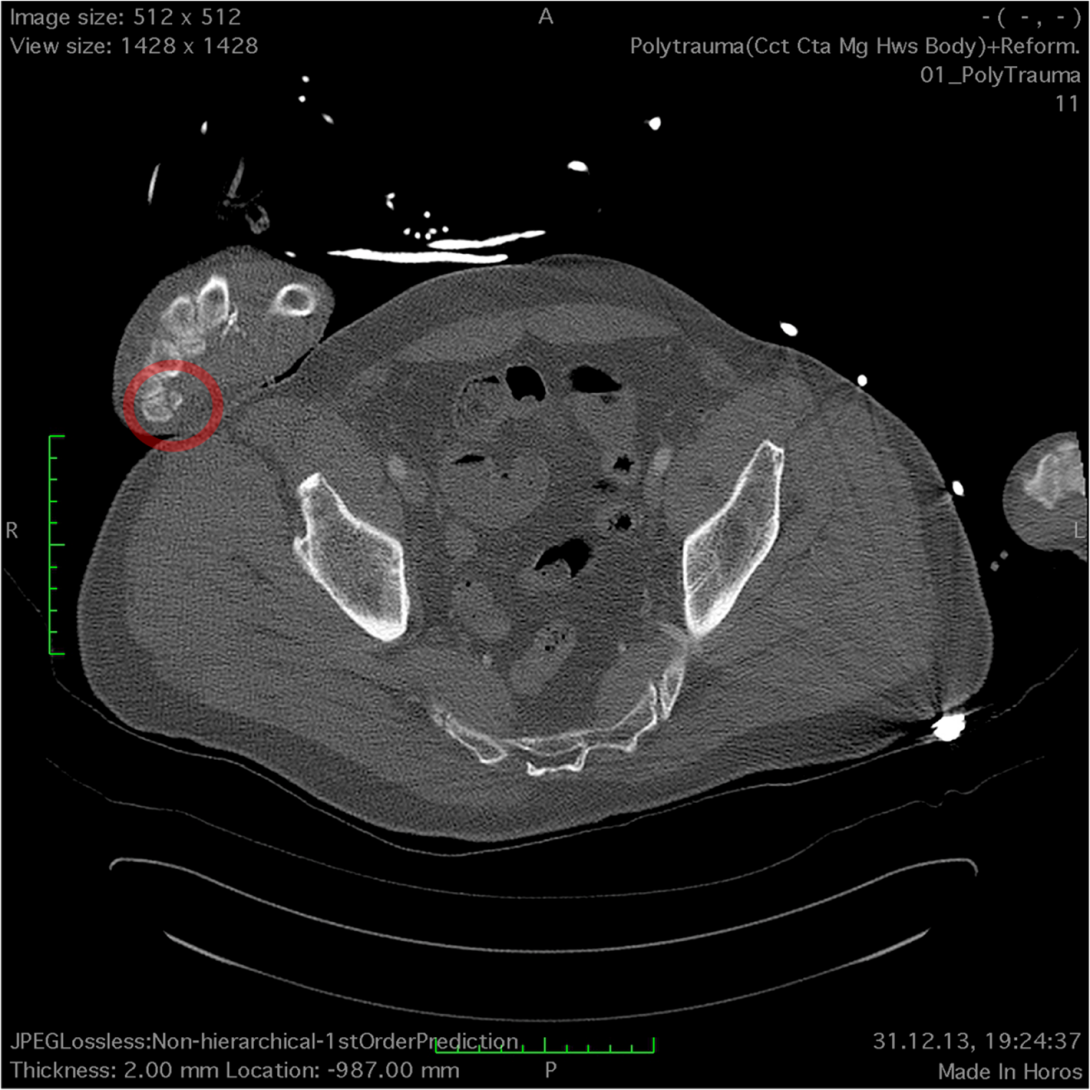
Fracture of the pisiform bone that was found on blinded reassessment. The adjacent slices are shown in the additional video file (additional file 1)

Radiologist 1 missed all reported carpal and phalangeal fractures but found a previously unknown carpal injury (pisiform fracture: Fig. 1 and Additional file 1).

**Fig. 2:**
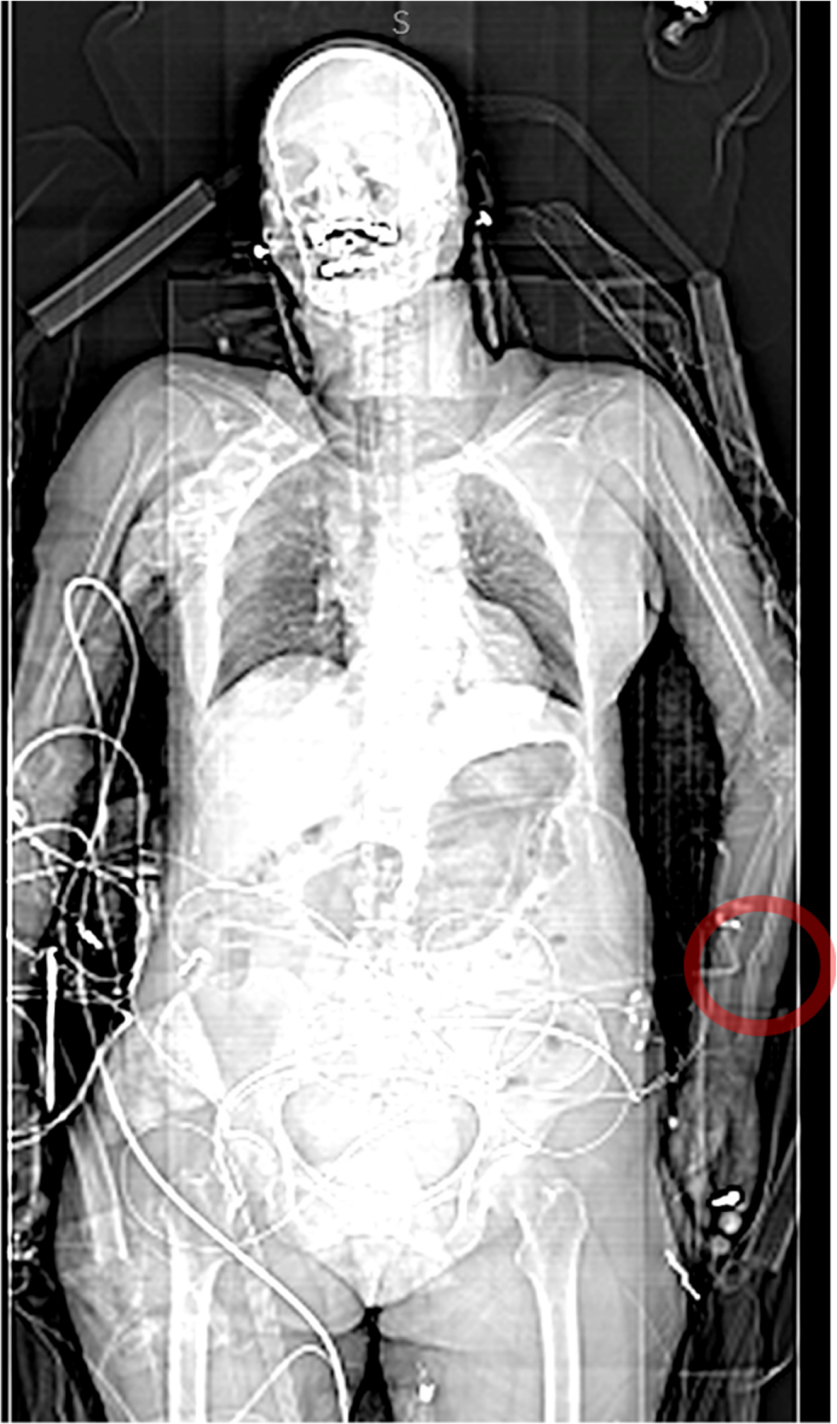
Fracture of the ulnar diaphysis that was only visible on the localiser as the arms were not included in the WBCT. Contrast and brightness were adjusted to better show the bones of the forearm

Eighteen known fractures were missed by both radiologists, 26 by Radiologist 1 only, and 4 by Radiologist 2 only. Of note is the use of the localiser by Radiologist 2 to look for an injury, as one ulnar fracture was only included there (Fig. 2).

This fracture was labelled visible in WBCT. Three new fractures of the ulna were of the styloid process in association with a distal radius fracture and might be considered not relevant by some doctors. The remaining four carpal fractures would be treated by a splint, the metacarpal fracture could be fixated by K-wire or splinted depending on possible malrotation as there was no angulation.

### Possible factors for missed/late diagnoses

On reassessment, all reported fractures were considered visible by both radiologists when confronted with the diagnosis. Reasons for missed fracture on reassessment included visibility in only one orientation, artifacts, and no reason. Two suspected fractures (scaphoid and metacarpal) on reassessment could not be confirmed or rejected and would have been followed up by additional imaging. They were not counted as fractures for this study.

**Fig. 3:**
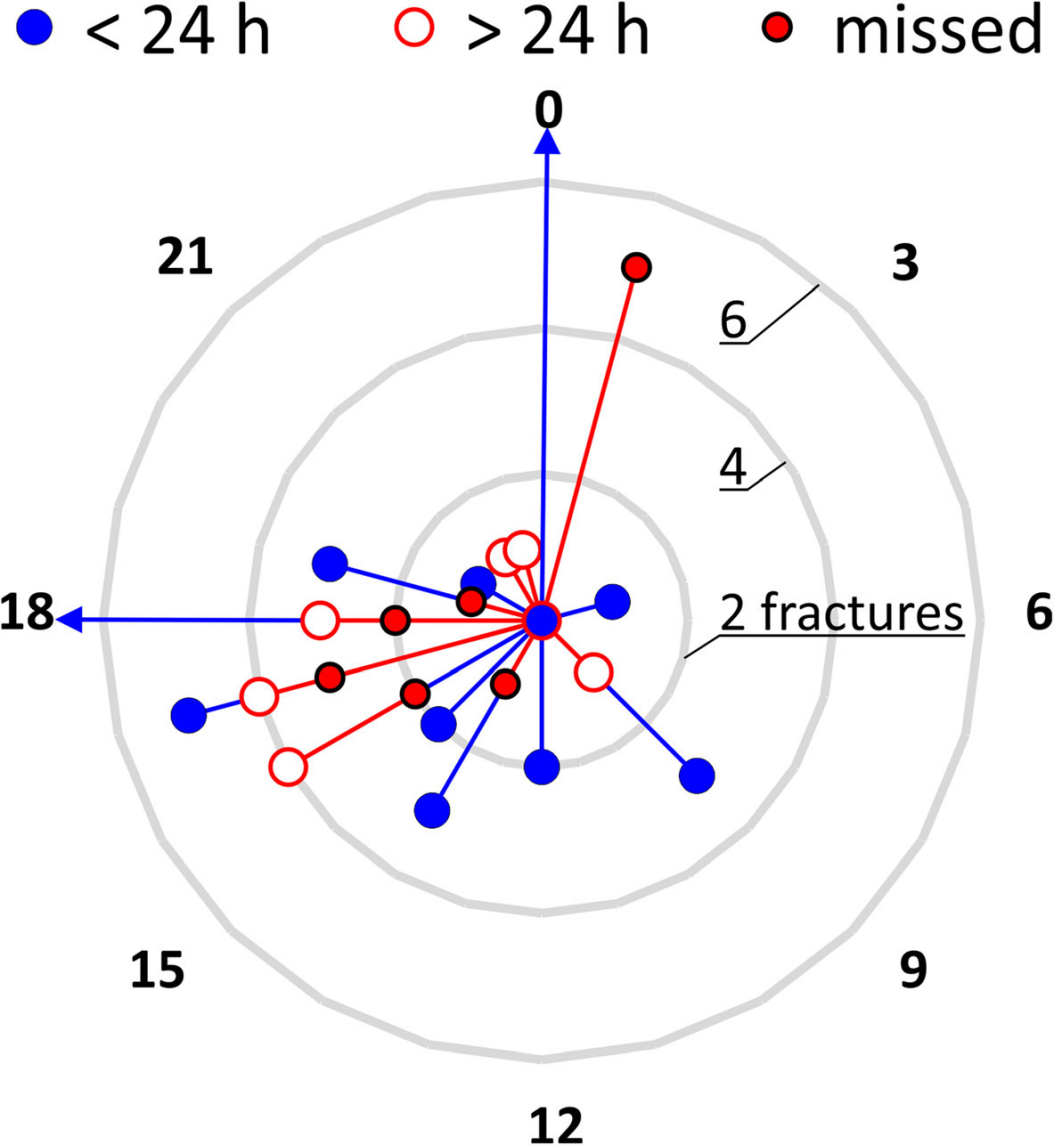
Number of fractures for time of the day of the WBCT. The circles show if the fracture was found within or after 24 h after submission or during this study (missed). The number of fractures found at 18 and 0 within 24 h were 14 and 9. The same diagram ist shown without fractures found within 24 h in additional file 2

Analysis of the time of day showed a tendency for missed or late diagnoses of potentially visible fractures for WBCT that were performed around 5 pm and 1 am (Fig. 3).

**Fig. 4:**
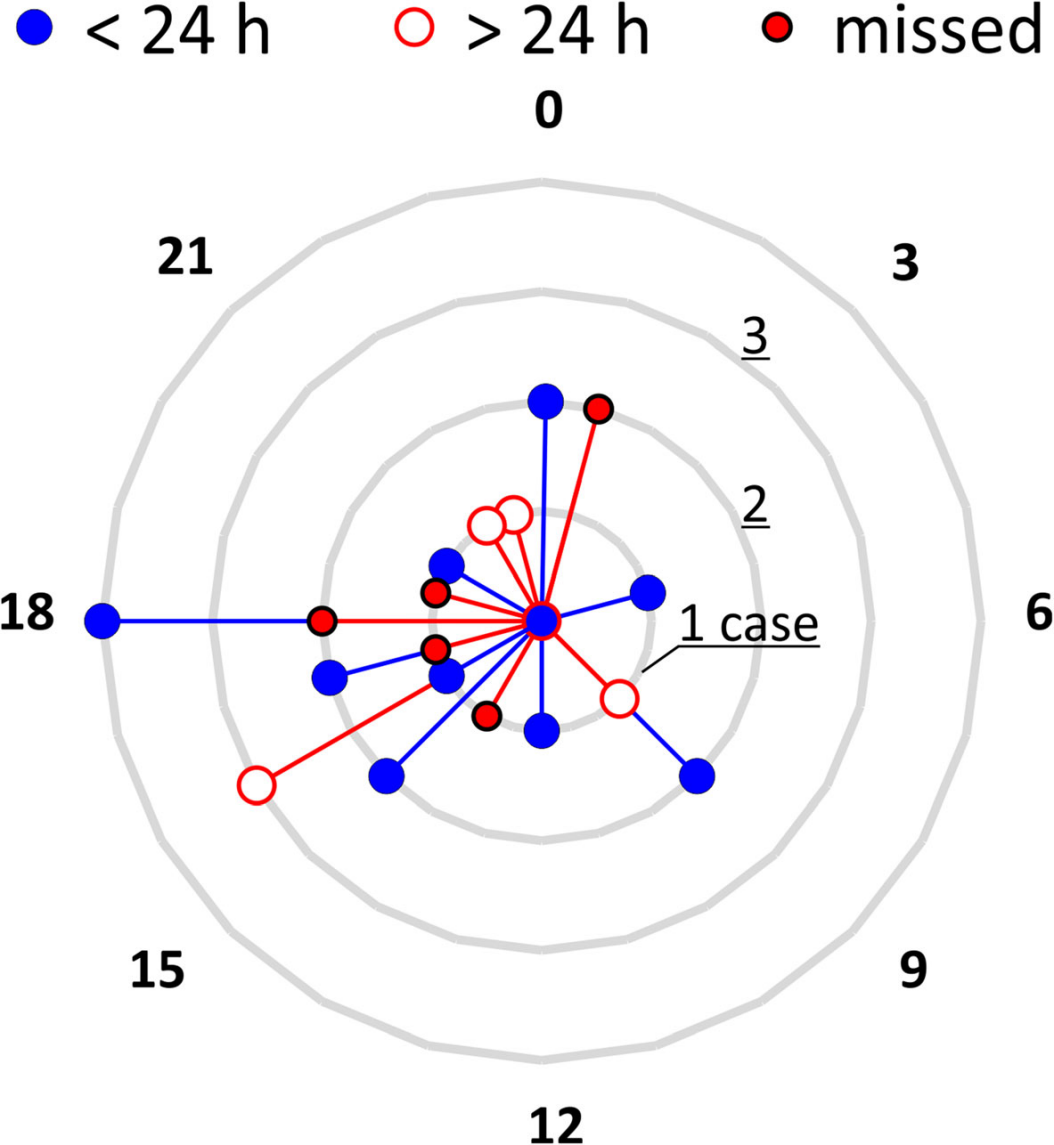
Number of cases with fractures for time of the day of the WBCT. The circles show if the fracture was found within or after 24 h after submission or during this study (missed). If at least one fracture was missed or found after 24 h, the label was set to missed or > 24 h. The same diagram ist shown without cases with fractures found within 24 h in additional file 3

Cases with at least one missed or late diagnosis of a fracture of hand or forearm showed a similar distribution (Fig. 4).

The same figures showing only the number of missed and late fractures and cases are shown in Additional files 2 and 3.

Slice thickness ranged between 0.9 and 5 mm with an average of 1.7 (CI 1.5–2.0, SD 1.1) for all 73 visible fractures.

Fractures that were diagnosed late or missed showed no difference in WBCT slice thickness to those found within 24 h (late/missed: *n* = 12, 1.7 mm, CI 1.1–1.2, SD 1.3 vs other *n* = 52, 1.7 mm, CI 1.5–2.0, SD 1.0, *p* = 0.743, t-test). The same was true looking at cases with late or missed fractures (late/missed: *n* = 12, 1.3 mm, CI 0.8–1.7, SD 0.7 vs other: *n* = 21, 1.8 mm, CI 1.4–2.3, SD 1.0, *p* = 0.092, t-test).

## Discussion

Fractures of forearm and hand in patients with multiple trauma can occur in 36% of cases [12]. In our original study population, we determined a prevalence of 12.1% for late diagnosis of fractures of forearm and hand [6]. In this subsample, we found 8 visible fractures in addition to 65 reported that were all visible in WBCT on reassessment. Missed fractures that were found during reassessment accounted for 11% of visible fractures in the WBCT. Even not counting three fractures of the ulnar styloid that were associated with a radius fracture, the remaining five fractures would add more than 7% that might not get proper treatment.

The treatment consequences of reported delayed diagnoses have been shown in our previous study [6]. In this study, one missed fracture might have needed a surgical treatment.

Previous studies reported a range of missed injuries up to 39%, corresponding to 65% of analysed patients who had a missed injury [3, 13, 14]. But they were eventually found during the treatment of the patient and not completely missed.

While both radiologists performed poorly in the reassessment of the WBCT as they missed more than 20 of 65 known fractures, but both found previously undetected fractures. One reason for the poor result might be motivation as they were doing the examination during breaks or after work. But that would not account for the number of new fractures that were found. Another explanation might be incomplete clinical data in the request forms. In both hospitals, the trauma surgeon would talk to the radiologist directly and discuss clinical signs that could hint to an injury. Depending on the patient’s condition, the one responsible for requesting the WBCT might not want to delay the procedure by writing a detailed essay. As little clinical data was available for the reassessment, both radiologists who reassessed the cases had few clues on possible injury areas, and they had to scan all areas with the same attention. In our sample, late and missed diagnoses cannot be attributed to a lower image quality regarding slice thickness.

Tertiary trauma survey can detect 56% of early missed injuries within 24 h [14]. The rate might be increased by addition of a radiological repeated survey along with the clinical examination.

In a systematic analysis of emergency radiographs for the extremities, the most common reason for a missed fracture was subtlety of the fracture [15]. The proposed solution was adequate training. Regarding our sample, the same might be true beginning with examination of all available data, including the localiser, and paying attention to hand and forearm when vital injuries have been excluded. While an experienced radiologist might have included inspection of the localiser in his routine, a less experienced viewer might forget it. The value of localiser examination was shown in a case series of abdominal CT scans that had a visible lung mass in the localiser that was not included in the axial sections and diagnosed several weeks later [16].

While not enough to be certain, we most missed fractures in our sample appeared late afternoon and shortly after midnight. In the participating centres that provided the cases, around 5 pm the first shift would end and the second would already have worked for several hours. Atypical working times may have a negative effect on psychomotor performance, lead to a higher risk of accidents and mood disturbances [17]. In addition, time of day was shown to matter for alerting attention in contrast to orienting and executive attention, the first being likely more important for assessment of radiographs [18]. Radiology reports were more often edited at end of shifts at 5 pm and with increasing working hours [19]. Fatigue and experience of radiologists has an influence on diagnostic efficiency and efficacy [20, 21]. Shift workers who worked into the night were shown to show a peak in the Karolinska Sleepiness Scale as the night advanced [22]. As we analysed a defined area and the radiologists missed fractures on reassessment without discernible pattern, a satisfaction of search effect cannot be excluded but is not likely in our study [23].

## Conclusions

The number of late diagnosis or completely missed fractures of the hand and forearm may be reduced by a repeated survey of WBCT with focus on the extremities.

## Supplementary Information

**Additional file 1.** Picture sequence combined to a video of an excerpt of the axial WBCT images. On the right patient wrist, a fracture of the pisiform bone can be seen that was found on blinded reassessment. The location of the fracture is marked in Fig. 1.

**Additional file 2.** Number of fractures for time of the day of the WBCT. The circles show if the fracture was found after 24 h after submission or during this study (missed). The same diagram is shown with fractures found within 24 h in Fig. 3.

**Additional file 3.** Number of cases with fractures for time of the day of the WBCT. The circles show if the fracture was found after 24 h after submission or during this study (missed). If at least one fracture was missed or found after 24 h, the label was set to missed or > 24 h. The same diagram is shown with cases of which all fractures were found within 24 h in Fig. 4.

## Data Availability

The dataset used and analysed during the current study is available on Mendeley Data: Münn, Friederike; Kim, Simon (2021), “Polytrauma: missed fractures hand/forearm”, Mendeley Data, V1, doi: 10.17632/f9vm3c4d7v.1

## Abbreviation

WBCT: Whole-body CT
