# Supplementary figures and images for: Detection of missed fractures of hand and forearm in whole-body CT in a blinded reassessment
Source: BMC Musculoskelet Disord. 2021 Jun 26;22:589. doi: 10.1186/s12891-021-04425-z (PMC8236191; doi:10.1186/s12891-021-04425-z)

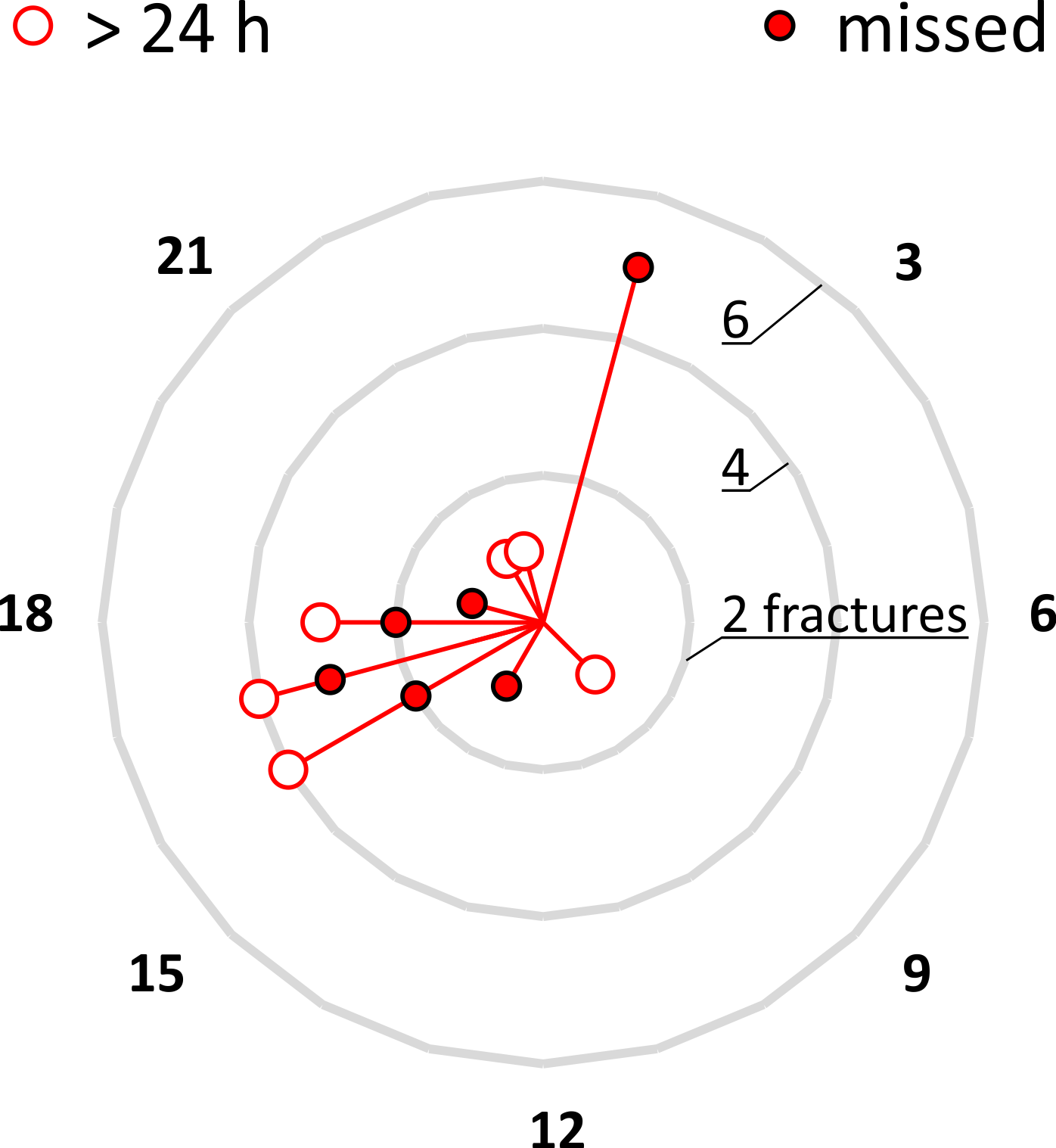

Supplement: Supplementary file 2 — Additional file 2. Number of fractures for time of the day of the WBCT. The circles show if the fracture was found after 24 h after submission or during this study (missed). The same diagram is shown with fractures found within 24 h in Fig. 3. [file 12891_2021_4425_MOESM2_ESM.png]

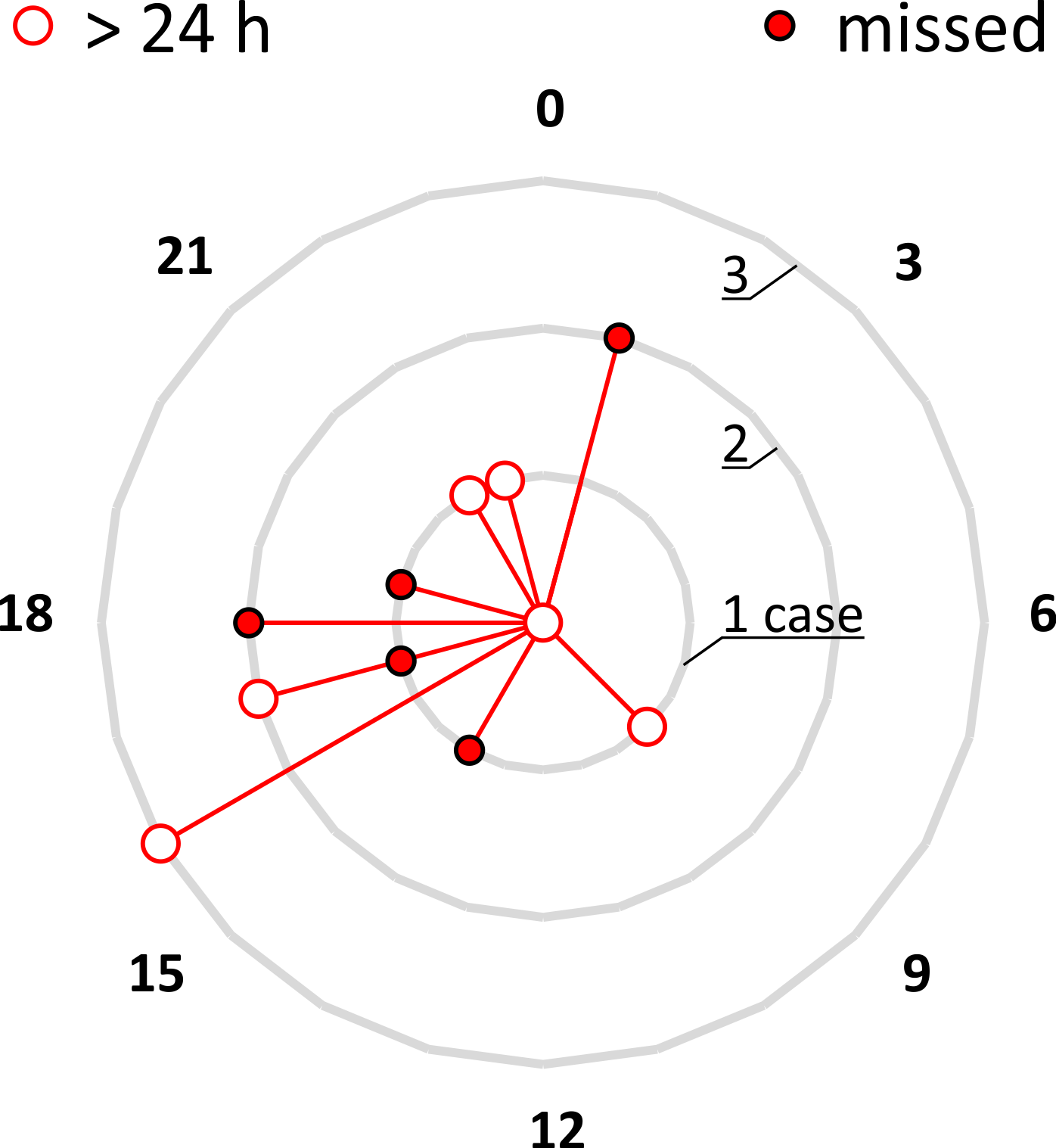

Supplement: Supplementary file 3 — Additional file 3. Number of cases with fractures for time of the day of the WBCT. The circles show if the fracture was found after 24 h after submission or during this study (missed). If at least one fracture was missed or found after 24 h, the label was set to missed or > 24 h. The same diagram is shown with cases of which all fractures were found within 24 h in Fig. 4. [file 12891_2021_4425_MOESM3_ESM.png]
